# Cross-cultural adaptation and validation of the Condom Self-Efficacy Scale: application to Brazilian adolescents and young adults ^1^

**DOI:** 10.1590/1518-8345.1062.2991

**Published:** 2018-01-08

**Authors:** Carla Suellen Pires de Sousa, Régia Christina Moura Barbosa Castro, Ana Karina Bezerra Pinheiro, Escolástica Rejane Ferreira Moura, Paulo César Almeida, Priscila de Souza Aquino

**Affiliations:** 2 MSc.; 3 PhD, Adjunct Professor, Departamento de Enfermagem, Universidade Federal do Ceará, Fortaleza, CE, Brazil.; 4 PhD, Adjunct Professor, Universidade Estadual do Ceará, Fortaleza, CE, Brazil.

**Keywords:** Condom, Validation Studies, Nursing

## Abstract

**Objective:**

translate and adapt the Condom Self-Efficacy Scale to Portuguese in the Brazilian context. The scale originated in the United States and measures self-efficacy in condom use.

**Method:**

methodological study in two phases: translation, cross-cultural adaptation and verification of psychometric properties. The translation and adaptation process involved four translators, one mediator of the synthesis and five health professionals. The content validity was verified using the Content Validation Index, based on 22 experts’ judgments. Forty subjects participated in the pretest, who contributed to the understanding of the scale items. The scale was applied to 209 students between 13 and 26 years of age from a school affiliated with the state-owned educational network. The reliability was analyzed by means of Cronbach’s alpha.

**Results:**

the Portuguese version of the scale obtained a Cronbach’s alpha coefficient of 0.85 and the total mean score was 68.1 points. A statistically significant relation was found between the total scale and the variables not having children (p= 0.038), condom use (p= 0.008) and condom use with fixed partner (p=0.036).

**Conclusion:**

the Brazilian version of the Condom Self-Efficacy Scale is a valid and reliable tool to verify the self-efficacy in condom use among adolescents and young adults.

## Introduction

The condom is a contraceptive barrier method that grants double protection, as it prevents pregnancy and sexually transmitted diseases (STDs). Therefore, it needs to be used consistently, that is, in all sexual relationships and correctly.

The inconsistent use of condoms among adolescents and young adults has been discussed in the literature and can be observed in different countries. In the Northwest of Cameroon, for example, the rate of volatile condom use among 414 interviewees was considered high (62%)^1^. In Brazil, according to data from the National School Health Survey, one in every four adolescents who started sexual life did not use a condom^2^.

Thus, the epidemiological data signal the need for sexual and reproductive health promotion strategies directed at adolescents and young adults. In addition, perceiving this population as prone to the non-use of this barrier method and knowing the main factors associated with this susceptibility are fundamental to guide the health professionals’ actions.

Self-efficacy associated with condom use is defined as the trust in one’s own capacity to practice safe sex in difficult situations^3^. This characteristic should be identified in order to prevent the main vulnerabilities and needs to be stimulated with a view to improving the young people’s compliance with condom use, in view of its influence on the use of the method. Self-efficacy influences condom use and can be stimulated among adolescents and young adults in order to increase compliance with the method^4^. 

In 1999, a North American nurse from Indiana University, USA developed the Condom Self-Efficacy Scale (CSE) to assess the perceived self-efficacy of condom use among adolescents and young adults. The CSE is a multifactorial tool that consists of 14 items that measure a set of cognitive and motivational skills, besides social and behavioral variables that reveal the self-efficacy in condom use. The factors analyzed are divided among the following domains: (1) communication skills related to condom use, with five items; (2) skills in consistent condom use with three items and (3) skills in consistent condom use with six items. These are measured on a five-point Likert scale, in which 1 represents very unsure, 2 unsure, 3 somewhat sure, 4 sure and 5 very sure^4^.

In view of the lack of adapted tools in Brazil to assess condom use, the CSE was chosen, as the self-efficacy concept influences the use of the method. In addition, the CSE included adolescents, which was very interesting as investigating this population is very important to support specific nursing interventions for this group. 

In that conjuncture, the aim in this study was to translate and adapt the CSE scale to Portuguese in the Brazilian context. A valid and reliable scale can be used in nursing practice and in other studies to assess interventions that improve the self-efficacy of adolescents and young adults to use a condom.

## Method

A methodological cross-cultural adaptation study was undertaken, in which the following method was adopted for the scale translation and adaptation process, in five phases, as follows: initial translation (1), consensus version of translations (2), back-translation (3), review by an expert committee (4) and pretest (5)^5^.

### Cross-cultural adaptation procedures

As a prerequisite to start the translation and cross-cultural adaptation process, the author of the scale was contacted by e-mail to present the research objectives. A translator who is a health professional and was knowledgeable about the objective of the translation translated the CSE (T1). A second translator did not come from the health area and was not informed about the study objective (T2). Both translations (T1 and T2) were combined in a consensus version (T12) to facilitate the decision on the synthesis of the items, taking strict care to maintain the sense of the original scale.

In the back-translation based on version T12, two American translators independently back-translated the scale to the original language. They lived in Brazil and mastered Portuguese and the Brazilian culture but had no experience in health and were not informed on the study objective.

The goal of the expert committee is to develop the pre-final version for the field test. It consisted of five professionals: three nurses, one translator and one linguist. The characteristics of the judges included: experience in sexual and reproductive health, translation and cross-cultural adaptation method of scales, mastery of English and Portuguese. Contact with the committee participants took place by e-mail. The following criteria were assessed: analysis of semantic, idiomatic, experiential and conceptual equivalence. Items with comprehension problems were modified by words that were better understood, making sure to maintain the meaning of the original items.

The objective of the pretest is to test the adapted scale and analyze the understanding of the scale by the target public. It was applied to a population of 40 subjects, in accordance with international recommendations. The participants in this phase were adolescents and young adults from 13 to 26 years of age, with an active sexual life and unfinished primary education, finished secondary education and finished higher education. They were contacted to apply the scale and instigated on the meaning of the items, as well as on contributions to improve the understanding of the scale. This guarantees that the adapted version maintains its equivalence in a practical context^5^. 

This step allowed the target public to significantly contribute to the validation process of the scale, which took place in September 2014 at a primary health care service, a technical school and a public university. 

### Verification of psychometric properties 

The criteria analyzed in this phase involved relevance, understanding and clarity, association of the item with the theme addressed, which domain the item refers to, relevance and degree of relevance of the item. After the expert committee had assessed the scale, the Content Validation Index (CVI) was used, which assesses the experts’ agreement with the content the scale represents, whose coefficient should be superior to 0.78 when more than six judges engage in the assessment^6^. It is added that, for the CVI, three mathematical equations were developed: mean convent validation index for all items in a scale, proportion of scale items that reached score 3 relevant or 4 highly relevant, by all judges, and content validation of the individual items^7^.

The construct validation of the CSE was done after the application of the scale to the target public based on the factor analysis. The sampling adequacy measure Kaiser-Meyer-Olkin (KMO) was used, as well as Bartlett’s sphericity test. Scores superior to 0.5 are recommended as acceptable, and scores of 0.7 or higher are considered good; scores between 0.8 and 0.9 are very good and superior to 0.9 excellent^8^.

In the factor analysis, the relation between the item and the factor is measured based on the factor loadings, which can range from -1.00 to + 1.00. The minimum factor loading should be 0.3, positive or negative, to indicate a relation between the item and the factor determined. In this study, when the factor loading was equal or superior to 0.3 and the item was allocated in the domain in question^9^. 

The internal consistency test Cronbach’s alpha was chosen to analyze the homogeneity of the CSE, the most common reliability measure. This test measures the one-dimensionality of a tool, with acceptable tools ranging from 0.7 to 0.8. Thus, when a tool has several sub-components (domains), the alpha should be calculated individually for each domain as, theoretically, various constructs exist^10^ ^-^ ^11^. 

### Application of information collection 

The construct validation took place at a state-owned school. The Brazilian version of the CSE was applied to 209 adolescents and young adults between 13 and 26 years of age, sexually active, regularly enrolled in the school affiliated with the state-owned education network of Ceará and located in the city of Fortaleza - CE. In addition, a questionnaire was applied to analyze the sociodemographic and sexual variables. 

For the sake of association, the chi-squared statistical tests were used, as well as Friedman’s test to compare the means among the domains. In all tests applied, significance was set as < 0.05. 

The study obtained a favorable opinion from the Research Ethics Committee at *Universidade Federal do Ceará* under protocol 702.946/2014. All subjects signed the free and informed consent form.

## Results

**Figure 1:**
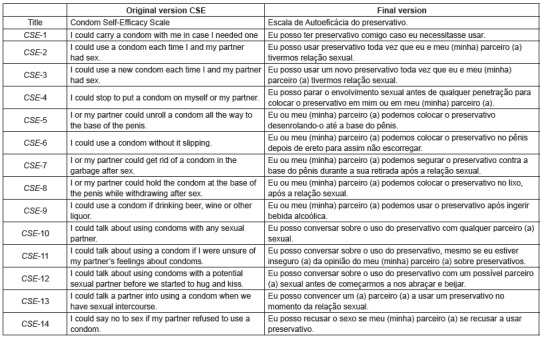
Translation and adaptation of the Condom Self-Efficacy Scale, 2014

Figure 1 displays the original and final version of the scale after the translation and cross-cultural adaptation process.

Twenty-two nurses were responsible for the validation of the scale contents, 86.3% of whom were women. As regards the activity area, 72.7% were active in research and teaching and 31.8% in teaching and care. The length of professional experience ranged between five and 29 years.

After the expert committee had assessed the items, items 7 and 8 were reorganized. All experts affirmed a more acceptable order with regard to the condom use. A CVI of 0.90 was obtained for the total scale, while the individual indices of the items ranged between 0.81 and 1, considering the scale contents valid.

### Construct Validity

The characteristics of the sample showed a slight male majority among the participants, who represented 50.7% of the adolescents and young adults. The predominant sexual option was heterosexual, referred by 89% of the participants. The age ranged between 13 and 26 years, the same age range adopted by the author of the original scale. As for the marital status, 66% had a partner and 9.1% had children.

What condom use with the fixed partner is concerned, 58.5% presented inconsistent use of the method. 

### Application of the Condom Self-Efficacy Scale - Brazilian version (CSE-VB)

The answers to the CSE-VB were analyzed by means of the total scale and domain scores: Communication, consistent use and correct used, as observed in Table 1.

**Table 1:** Association of total scale and communication, consistent condom use and correct condom use domain scores with sociodemographic and sexual characteristics of students. Fortaleza, CE, Brazil, 2014

Sociodemographic and sexual characteristics			N	Mean	SD*	p^†^
Total scale						
	Sex					0.350
		Male	106	69.4	23.9	
		Female	103	66.7	22.7	
	Marital status					0.341
		With partner	138	67.1	21.4	
		Without partner	71	70	18.4	
	Children					0.038
		Yes	19	58.8	20.2	
		No	190	69.0	21.1	
	Condom use with fixed partner (n=123)					0.036
		Never	22	57.1	25.2	
		Hardly	19	67.5	21.3	
		Sometimes	31	64.1	22.1	
		In most relations	21	72.2	19.5	
		In all relations	30	75.2	17.3	
Domain 1: Communication						
	Sex					0.277
		Male	106	69.1	22.7	
		Female	103	72.7	25.2	
	Marital status					0.718
		With partner	138	71.3	24.5	
		Without partner	71	70.0	23.1	
	Children					0.040
		Yes	19	60	23.4	
		No	90	71.9	23.9	
	Condom use with fixed partner (n=123)					0.077
		Never	22	66.6	28.3	
		Hardly	19	66.2	23.9	
		Sometimes	31	69.8	20.6	
		In most relations	21	74.2	27.1	
		In all relations	30	82.6	19.8	
Domain 2: Consistent use						
	Sex					0.028
		Male	106	70.5	22.4	
		Female	103	62.8	27.7	
	Marital status					0.201
		With partner	138	65.1	26.7	
		Without partner	71	69.8	22.5	
	Children					
		Yes				
		No				
	Condom use with fixed partner (n=123)					0.003
		Never	22	48.6	32.4	
		Hardly	19	70.0	21.6	
		Sometimes	31	59.3	27.7	
		In most relations	21	73.5	19.3	
		In all relations	30	73.8	25.2	
Domain 3: Correct use						0.066
	Sex					
		Male	106	68.3	23.9	
		Female	103	61.5	29.3	
	Marital status					0.042
		With partner	138	62.3	28.0	
		Without partner	71	70.3	23.6	
	Children					0.490
		Yes	19	60.9	27.7	
		No	190	65.4	26.8	
	Condom use with fixed partner (n=123)					0.687
		Never	22	54.9	27.6	
		Hardly	19	66.2	30.6	
		Sometimes	31	60.4	33.4	
		In most relations	21	66.2	27.1	
		In all relations	30	63.0	35.6	

*Standard Deviation; †Chi-Squared Test

Friedman’s test was significant (p=0.0321), showing that significant differences existed between the mean domain scores. The analysis of the mean score of the total scale resulted in 68.1 points, showing that the students felt secure to use a condom, that is, they presented self-efficacy in the situations addressed in each scale item. 

Concerning the association between the sociodemographic and sexual variables and the scale domains, in the communication domain, the fact of having children influences the negotiation about the condom use (p= 0.040).

The domain consistent condom use contained questions related to condom use in all sexual relations and the ease to use the method. A statistically significant relation exists between the mean CSE in the domain consistent condom use and the variables sex (p=0.028), condom use (p=0.002) and consistent condom use with fixed partner (p=0.003). The male sex presented greater self-efficacy in condom use. 

Having a fixed partner was associated with the mean scores in the consistent use and correct use domains (p=0.020). Another variable associated with the mean coefficient in the correct use domain was not having a partner (p=0.042). This domain involved the questions related to the steps recommended in the literature about condom use and the degree of security to take each step. 

The KMO coefficient in this study corresponded to 0.862 and the sphericity test demonstrated statistical significance (p = 0.001). Therefore, these results revealed that the factor analysis is appropriate to analyze the CSE^8^.

The Cronbach’s alpha coefficient for each domain of the scale were calculated individually, which ranged from 0.632 to 0.788.

**Figure 2:**
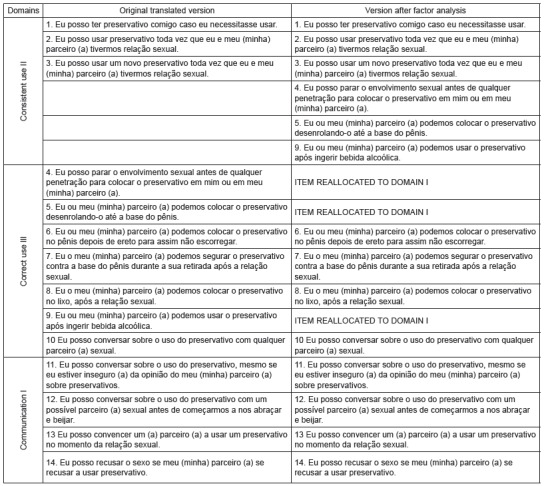
Comparison between original scale and version after factor analysis.

Figure 2 shows a comparison between the translation of the original scale and the version after the factor analysis, when some items were reallocated.

As observed, items 4, 5 and 9 were reallocated to another domain after the factor analysis. The result does not minimize the scores of the scale, as no item needed to be removed, but simply reallocated.

## Discussion

In the adaptation process, slight changes were made, such as adding the female sex among the research possibilities, in order to include this sex in the condom use. In item 4, the inclusion of the term “sexual relation” was suggested, which contributed to make the item clearer and more understandable. In addition, replacing the word “potential” by “possible” was suggested in item 12. This change was pertinent, as the term “potential” can refer to a characteristic of the partner, while the word “possible” indicates the possibility that a sexual partner might come up.

In item 11, the linguist suggested replacing the word “feelings” by “opinion”. It is highlighted that the linguist’s presence was fundamental to correct the verbal tenses, enriching the grammatical context. In the content validation, the 22 experts on the committee approved the final version, appointing the relevance of the scale in the nursing context.

The application of the Brazilian version of the CSE identified that the fact of not having children is associated with the adolescents and young adults’ self-efficacy in the communication domain, which involves self-efficacy for the will to use a condom with one’s partner. Thus, empowerment to use the method is essential to argue with one’s partner^12^ ^-^ ^13^.

The male sex showed greater self-efficacy in terms of consistent condom use. This finding supports the evidence appointed in a study that involved 508 male and female participants in Uganda. When comparing the consistent condom use between men and women, the score for men was 48.1%, against 31.8% for women^14^. This difference according to sex involves cultural aspects, as men are stimulated early to have sexual relations, while women are discouraged.^15^. Therefore, men dominate the use of the method, contributing to the higher percentage, while women are confronted with people’s judgments on the capacity to execute certain activities and achieve certain kinds of performance, that is, the practice of condom use predisposes their self-efficacy^16^. 

In addition, self-efficacy was also associated with the consistent condom use domain, concerning condom use with one’s fixed partner in all or most sexual relations. During a field study involving 8,471 adolescents from the state of Paraíba, in 31% of the reports, great female difficulty to negotiate on the condom was observed^17^. This reality was also found in a Brazilian cross-sectional study involving 17,371 secondary school students, in which unsafe sex was found among older girls with low socioeconomic conditions^18^. 

It is inferred that adolescents and young adults who use a condom with their fixed partner present high self-efficacy scores. This finding differs from another study in which inconsistent condom use was observed among young people with fixed partners, as many tend to abandon the method as the relationship gains stability^19^. 

Having a fixed partner influences correct condom use (p=0.020). Hence, self-efficacy should be encouraged in this population, as difficulties with correct condom use imply the abandonment of the method. A study involving 166 adolescents showed that this population faces difficulties to correctly use the condom. Among the adolescents interviewed, 16.9% indicated difficulty to use the method in their first sexual relation^20^. The difficulties in this face of sexual discovery induce the non-use of the method and the adoption of sexual risk behaviors. 

Concerning the construct validation using factor analysis, three domains were extracted, the same number as in the original scale. It is highlighted that the reallocation of item 9 to the communication domain was also evidenced in the adaptation process of the CSE in Thailand, where the item presented an index superior to 0.3 in the communication domain as well as in the correct use domain. Hence, it is inferred that the item mentioned can influence both the ability to communicate on the condom use and the correct use of the method^21^. 

The scale presented good internal consistency, with a Cronbach’s alpha coefficient (0.85) similar to the original scale and the adapted version for Thailand. In the adapted version for Korea, the alpha coefficient was superior (0.91)^4^ ^,^ ^21^ ^-^ ^22^. These data indicated the good reliability of the scale in different contexts. 

 It was noticed that the self-efficacy for condom use can be stimulated among students through educational interventions. The use of valid and reliable tools can help to identify individual vulnerabilities early.

## Conclusion

The use of the method described permitted a strict cross-cultural adaptation process of the CSE. The scale version after the assessment by the first expert committee and the application of the pretest was considered understandable to the target public. 

Thus, it can be concluded that the CSE-VB is a valid and reliable tool to measure the self-efficacy for condom use among adolescents and young adults. The Brazilian version of the CSE can be used in nursing practice to assess interventions that improve the self-efficacy for condom use.

It is highlighted that, even after a strict adaptation and validation process, for the use of this scale in other Brazilian regions, a new semantic validation process is necessary due to linguistic variations specific to each region.
